# Long non-coding RNA SDCBP2-AS1 delays the progression of ovarian cancer via microRNA-100-5p-targeted EPDR1

**DOI:** 10.1186/s12957-021-02295-2

**Published:** 2021-07-04

**Authors:** Xiu Liu, Chanyuan Liu, Aijun Zhang, Qi Wang, Jiao Ge, Qunying Li, Jinlei Xiao

**Affiliations:** Wuhan Wuchang Hospital, Wuhan University of Science and Technology, 505 Luoshi Road, South District, Wuchang Hospital, Hongshan District, Wuhan, 430061 Hubei China

**Keywords:** Ovarian cancer, Long non-coding RNA SDCBP2-AS1, MicroRNA-100-5p, Ependymin-related protein 1, Viability, Apoptosis, Migration, Invasion

## Abstract

**Background:**

Dysregulation of long non-coding RNAs has been implied to connect with cancer progression. This research was to decipher the mechanism of long non-coding RNA SDCBP2-AS1 in ovarian cancer (OC) through regulation of microRNA (miR)-100-5p and ependymin-related protein 1 (EPDR1).

**Methods:**

LncRNA SDCBP2-AS1 and EPDR1 levels in OC were assessed by Gene Expression Profiling Interactive Analysis. lncRNA SDCBP2-AS1, miR-100-5p, and EPDR1 levels in OC tissues and cells were determined. SKOV3 and A2780 cells were transfected with lncRNA SDCBP2-AS1, miR-100-5p, and EPDR1-related plasmids or sequences, and then their functions in cell viability, apoptosis, migration, and invasion were evaluated. The interplay of lncRNA SDCBP2-AS1, miR-100-5p, and EPDR1 was clarified.

**Results:**

LncRNA SDCBP2-AS1 and EPDR1 levels were suppressed whilst miR-100-5p level was elevated in OC. After upregulating lncRNA SDCBP2-AS1 or EPDR1, viability, migration, and invasion of OC cells were impaired, and apoptosis rate was increased. Downregulating EPDR1 or upregulating miR-100-5p partially mitigated upregulated lncRNA SDCBP2-AS1-induced impacts on the biological functions of OC cells. LncRNA SDCBP2-AS1 sponged miR-100-5p, and EPDR1 was targeted by miR-100-5p.

**Conclusion:**

It is illustrated that lncRNA SDCBP2-AS1 regulates EPDR1 by sponge adsorption of miR-100-5p to inhibit the progression of OC.

## Background

Ovarian cancer (OC) begins when normal cells in the ovaries are out of control to form tumors, and epithelial OC (EOC) accounts for 85–90% of OC cases [1]. Reproductive history, exogenous hormone use, medical history, and other benign gynecological conditions are all linked with the epidemiology of EOC [2]. Owing to lack of definite screening techniques, and vague signs and symptoms, early recognition and diagnosis are still the obstacles for the cure and survival of patients with OC [3]. Some pharmatherapeutical agents have developed to improve the prognosis of OC patients, such as poly ADP-ribose polymerase (PARP) inhibitors [4]. Besides, cytoreductive surgery and platinum/taxane combination chemotherapy are preferentially recommended to patients with OC; however, relapse and chemoresistance happen with dismal survival rate [5]. Thus, a thorough awareness of OC-related mechanism is crucial to formulating effective treatment strategies.

Long non-coding RNAs (lncRNAs) have been widely discussed in OC, as to regulation of cell fate and drug resistance, and evaluation of diagnosis and prognosis [6]. As a matter of fact, OC cells are moving to an uncontrolled state, in which aberrant overexpression of some lncRNAs perform critically, such as plasmacytoma variant translocation I PVT1 [7], metastasis-associated lung adenocarcinoma transcript 1 [8], and P73 antisense RNA 1T [9]. Regarding lncRNA SDCBP2-AS1, the update laboratory has only recorded its downregulation in thyroid cancer [10] while its functional mechanism has not been revealed in OC yet. LncRNAs-microRNAs (miRNAs) network in OC could regulate cancer development. For example, lncRNA mortal obligate RNA transcript could depress OC cell proliferation through suppression of miR-21 [11]. Also, forkhead box protein A1 downregulates miR-100-5p, thereby inhibiting the malignant phenotype of nasopharyngeal carcinoma cells [12]. In fact, aberrant expression of miRNAs is common in OC, and they are associated with clinicopathological features of OC patients [13]. Various oncogenic miRNAs have been listed in the course of OC, such as metastasis-related miR-552 [14] and drug resistance-related miR-1307 [15]. Moreover, it is well-known that regulating the deregulated miRNA could suppress the growth of OC, such as overexpression of miR-29c-3p [16]. miR-100-5p shows overexpression in ovarian endometriotic stromal cells [17]. High miR-100-5p presents prognostic values in unfavorable oral squamous cell carcinoma (OSCC) [18], and inhibition of it could lock cell activities in prostate cancer [19] and renal cell carcinoma (RCC) [20]. However, a vacancy for a comprehensive understanding of lncRNA SDCBP2-AS1 and miR-100-5p in OC is stood. Through bioinformatics website, it was predicted that miR-100-5p had a binding site with ependymin-related protein 1 (EPDR1). EPDR1 is a relatively uncharacterized protein in the lysosome and secretome of most vertebrates [21]. EPDR1 is commonly regarded to suppress tumor growth in human cancers, such as breast cancer [22]. But, its performance in OC has not been specified yet.

Our research aimed to figure out whether the regulatory link existed between lncRNA SDCBP2-AS1, miR-100-5p, and EPDR1, and even whether lncRNA SDCBP2-AS1/miR-100-5p/EPDR1 axis could mediate the cellular progression of OC. This study focusing on lncRNA SDCBP2-AS1/miR-100-5p/EPDR1 axis may provide the novel effective biomarkers for OC.

## Methods and materials

### Ethics statement

Informed consent was provided by all patients. The ethics committee of Wuhan Wuchang Hospital, Wuhan University of Science and Technology, signed an approval for the experiment.

### Collection of clinical tissues

Samples (n = 71) of cancer tissues and adjacent tissues (≥ 5 cm from the edge of the tumor) were harvested from OC patients (35–60 years old) in Wuhan Wuchang Hospital, Wuhan University of Science and Technology, and preserved in liquid nitrogen at −80°C. Patients were included if they underwent primary surgery, with complete data of preoperative chemotherapy and medical history. Otherwise, patients were excluded. Also, patients with other tumors were excluded.

### Cell culture

OC cell lines (COC1, A2780, and SKOV3) and normal ovarian epithelial cells (IOSE80) from ATCC (VA, USA) were placed in a culture system of Roswell Park Memorial Institute (RPMI) 1640 medium, 10% fetal bovine serum (FBS), and penicillin and streptomycin (100 μL/mL).

### Cell transfection

Cells (2 × 10^5^ cells/well) in 6-well plates were grown to 70% confluence and cultured in serum-free culture medium for 1 h. Cell transfection was conducted via Lipofectamine 2000 (Invitrogen, CA, USA). pcDNA3.1-lncRNA SDCBP2-AS1, pcDNA3.1-negative control (NC), pcDNA3.1-EPDR1, pcDNA3.1-control (CTR), si-EPDR1, miR-100-5p mimic, or mimic NC (50 nM) were accessible to GenePharma (Shanghai, China).

### 3-(4, 5-dimethylthiazol-2-yl)-2, 5-diphenyltetrazolium bromide (MTT) assay

Cells (3 × 10^5^ cells/mL) were firstly cultured for 48 h in 96-well plates, then combined with MTT solution (50 μL/well, 5 mg/mL) and kept for 4 h (a blank well without cells was set). After that, dimethyl sulfoxide (150 μL/well) was supplemented. Absorbance values at 450 nm were detected with a microplate reader.

### Flow cytometry

Cells (5.0 × 10^5^ cells/well) were processed by detachment with 0.25% trypsin (without ethylene diamine tetraacetic acid), centrifugation, and suspension in 1× binding buffer (100 μL). Then, the cell suspension was reacted with fluorescein isothiocyanate (FITC) Annexin V (5 μL) in the dark. As soon as propidium iodide (PI; 5 μL) was added, cells were supposed to be detected by flow cytometry within 1 h. Annexin V-FITC/PI kit was from BD Company (NJ, USA).

### Transwell assay

Cells after trypsinization were prepared into 1 × 10^5^ cells/mL with a serum-free cell culture medium. Matrigel (BD Bioscience, NJ, USA) was added to the Transwell chamber (Corning, NY, USA) for 3 h and solidified. Cell culture medium without FBS was placed into the chamber. Then, an appropriate amount of cell suspension was supplemented in the Transwell chamber (cell culture medium in the outside of the chamber). After incubating for 48 h, cells not penetrated the membrane were wiped off with a cotton swab, and others were fixed with paraformaldehyde, stained with crystal violet, and observed under a microscope. Matrigel was not used in the migration experiment.

### Reverse transcription quantitative polymerase chain reaction (RT-qPCR)

Total RNA extracted from tissues and cells via TRIzol kit (Tiangen Biotech Co., Ltd., Beijing, China). The RNA reverse transcription kit (Takara, Dalian, China) was adopted in RNA transformation into cDNA. LncRNA SDCBP2-AS1 and EPDR1 were applied glyceraldehyde-3-phosphate dehydrogenase (GAPDH) while miR-100-5p took U6 as the internal control. PCR primers (Table 1) were provided by Takara. After PCR amplification, the product was verified by electrophoresis on agarose gel. Ct value (threshold cycle) was obtained after manually setting the threshold value at the lowest point of the parallel rise of each logarithmic amplification curve. Data evaluation was performed by the 2^−ΔΔCt^ method.

**Table 1:** Primer sequences

Genes	Primers
miR-100-5p	Forward: 5′-ACGTCGTCATGGGGTACCCCA-3′
	Reverse: 5′-GTACGATCGATGCGCTACGTCG-3′
U6	Forward: 5′-ACTGATCGATGCCTGATCGATCG-3′
	Reverse: 5′-AAAGCTGTCCCGGGGTACGTGCC-3′
SDCBP2-AS1	Forward: 5′-TAAGAAACGGGTGGGGGTTG-3′
	Reverse: 5′-AATGCATACCCCAGCTCACC-3′
EPDR1	Forward: 5′-TGAAACCTGGATTGGCATCTATAC-3′
	Reverse: 5′-TGTAGTTTATGGTAAAGGTTTCCTG-3′
GAPDH	Forward: 5′-GACAACAGCCTCAAGATCATCAG-3′
	Reverse: 5′-GTGGCAGTGATGGCATGGA-3′

Note: *miR-100-5p* microRNA-100-5p, *SDCBP2-AS1* long non-coding RNA SDCBP2-AS1, *EPDR1* ependymin-related protein 1, *GAPDH* glyceraldehyde phosphate dehydrogenase

### Western blot assay

With the protein extraction kit (Thermo Fisher Scientific, IL, USA), total protein was extracted and quantified by bicinchoninic acid kit (Thermo Fisher). Electrophoresis was conducted with 10% separating gel and 4% concentrated gel. Protein (40 μg/well) after denature was transferred onto a polyvinylidene fluoride membrane via semi-dry method, and blocked with 5% skimmed milk powder. Following reaction with primary antibodies EPDR1 (1:1 000, Santa Cruz Biotechnology, CA, USA) and GAPDH (1:1 000, Abcam), the membrane was further probed with the secondary antibody (1:1500, Lincoln, NE, USA), developed, exposed, and analyzed with GAPDH as internal control.

### RNA immunoprecipitation (RIP) assay

RIP was performed with Magna RIP kit (Millipore, MA, USA). Cells were lysed by complete RIP lysis buffer and bound to the magnetic beads. Subsequently, the magnetic beads were combined with human anti-Ago2 or normal mouse immunoglobulin G (Millipore) for 24 h and treated with proteinase K. Immunoprecipitated RNA was subjected to RT-qPCR analysis.

### Dual luciferase reporter gene assay

The target sequence was predicted by the bioinformatics website, and lncRNA SDCBP2-AS1 or EPDR1 3′-UTR sequence binding to miR-100-5p was amplified and mutated via site-directed mutagenesis kit (NBS, Beijing) to generate lncRNA SDCBP2-AS1 or EPDR1 3′-UTR mutant (MUT) sequence. Subsequently, the amplified lncRNA SDCBP2-AS1 or EPDR1 3′-UTR sequence and the lncRNA SDCBP2-AS1 or EPDR1 3̲′-UTR MUT sequence were inserted into the psi-CHECK2 reporter (Promega, WI, USA) and sequenced. Wild-type (Wt)-lncRNA SDCBP2-AS1 or Wt-EPDR1 3′-UTR and the Mut-lncRNA SDCBP2-AS1 or Mut-EPDR1 3′-UTR vectors were collected. Wt-lncRNA SDCBP2-AS1 or Wt-EPDR1 3′-UTR vector, or Mut-lncRNA SDCBP2-AS1 or Mut-EPDR1 3′-UTR vector, and miR-100-5p mimic or mimic-NC were co-transfected into cells via Lipofectamine^TM^ 2000. The samples were harvested 48 h after transfection. Finally, the relative luciferase activity of firefly and Renilla was detected with the dual luciferase detection kit (Promega).

### Statistical analysis

All data were processed by SPSS 21.0 (IBM, NY, USA). Measurement data were presented as mean ± standard deviation. Except for the comparison of OC tissues and adjacent tissues by paired t test, the other two groups were compared by independent sample t test. One-way analysis of variance (ANOVA) was applied to multi-group comparison while Tukey’s multiple comparisons test to pairwise comparison. Pearson correlation analyzed the correlation between lncRNA SDCBP2-AS1 and EPDR1 in OC tissues. *P* < 0.05 was informed of statistical difference.

## Results

### Lowly expressed lncRNA SDCBP2-AS1 and EPDR1 present in OC

**Fig. 1:**
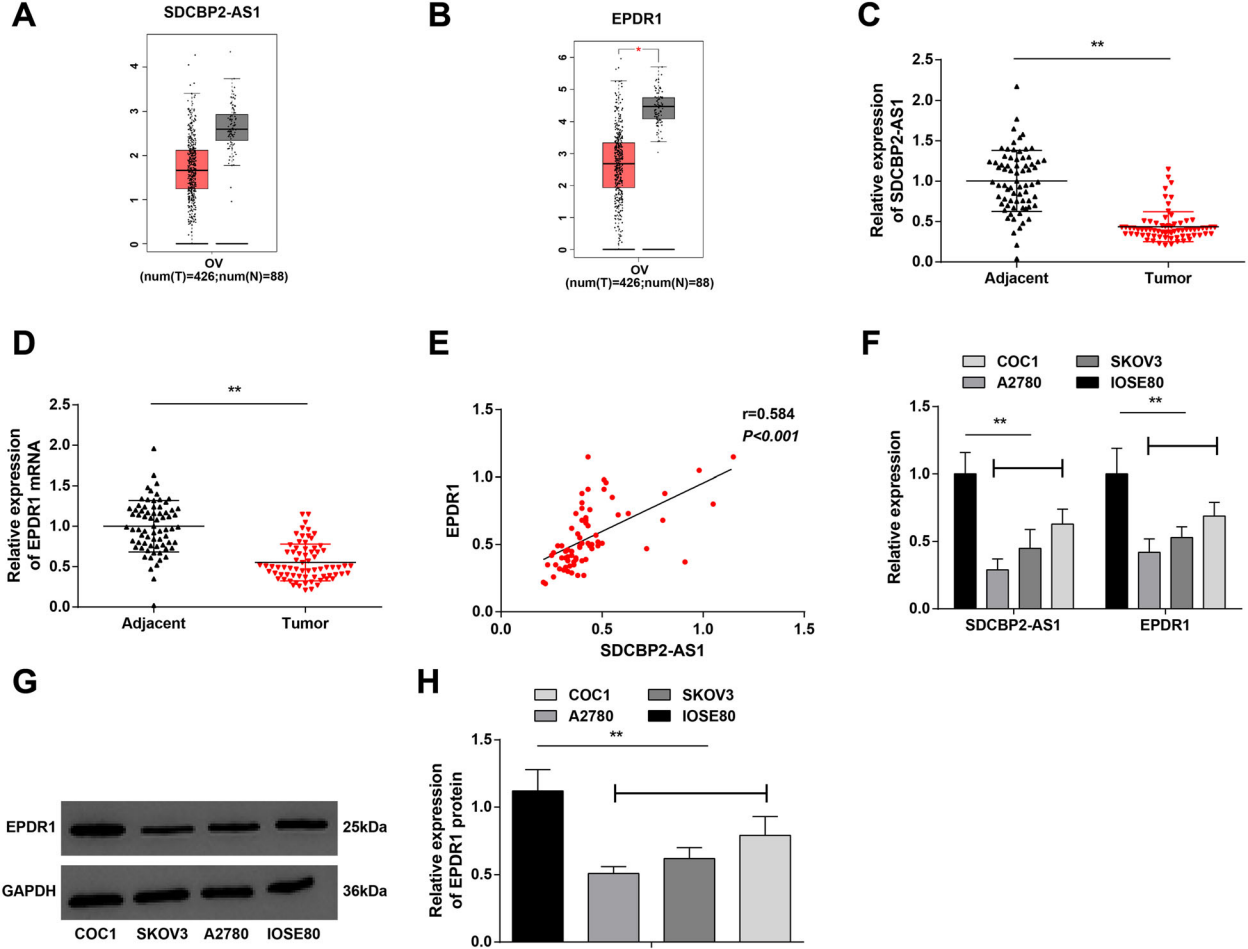
Lowly expressed lncRNA SDCBP2-AS1 and EPDR1 present in OC. **A** LncRNA SDCBP2-AS1 expression in OC tissues reflected by GEPIA website creator; **B** EPDR1 expression in OC tissues reflected by GEPIA website creator; **C** RT-qPCR detection of lncRNA SDCBP2-AS1 in OC tissues and adjacent tissues (*n* = 71); **D** RT-qPCR detection of EPDR1 in OC tissues and adjacent tissues (*n* = 71); **E** correlation between lncRNA SDCBP2-AS1 and EPDR1 levels in OC tissues reflected by Pearson correlation analysis (*n* = 71); **F** RT-qPCR detection of lncRNA SDCBP2-AS1 and EPDR1 in OC cell lines (COC1, A2780, and SKOV3) and normal ovarian epithelial cells (IOSE80), repetition = 3; G/H. Western blot analysis of EPDR1 in OC cell lines and IOSE80, repetition = 3; * *P* < 0.05, ** *P* < 0.01. Data were expressed in the form of mean ± standard deviation and analyzed by t test, one-way analysis of variance, and Tukey’s multiple comparison test

GEPIA website (http://gepia.cancer-pku.cn/) evaluated lncRNA SDCBP2-AS1 and EPDR1 mRNA expression in OC and found that they were both downregulated in OC (Fig. 1A, B). After that, 71 cases of OC tissues and adjacent tissues were utilized to measure lncRNA SDCBP2-AS1 and EPDR1 expression via RT-qPCR, which finally presented downregulation trend in OC tissues (Fig. 1C, D). Also, Pearson correlation analysis revealed a positive correlation between lncRNA SDCBP2-AS1 and EPDR1 levels in OC tissues (Fig. 1E). Moreover, lncRNA SDCBP2-AS1 and EPDR1 levels were examined in OC cell lines (COC1, A2780, and SKOV3) and normal ovarian epithelial cells (IOSE80) through RT-qPCR and Western blot, and their manifestations were consistent with those in OC tissues (Fig. 1F–H). Simply, lncRNA SDCBP2-AS1 and EPDR1 were downregulated in OC, and their expression levels in OC tissues were positively correlated. lncRNA SDCBP2-AS1 and EPDR1 may be involved in OC.

### Upregulating lncRNA SDCBP2-AS1 or EPDR1 suppresses OC cell development

**Fig. 2:**
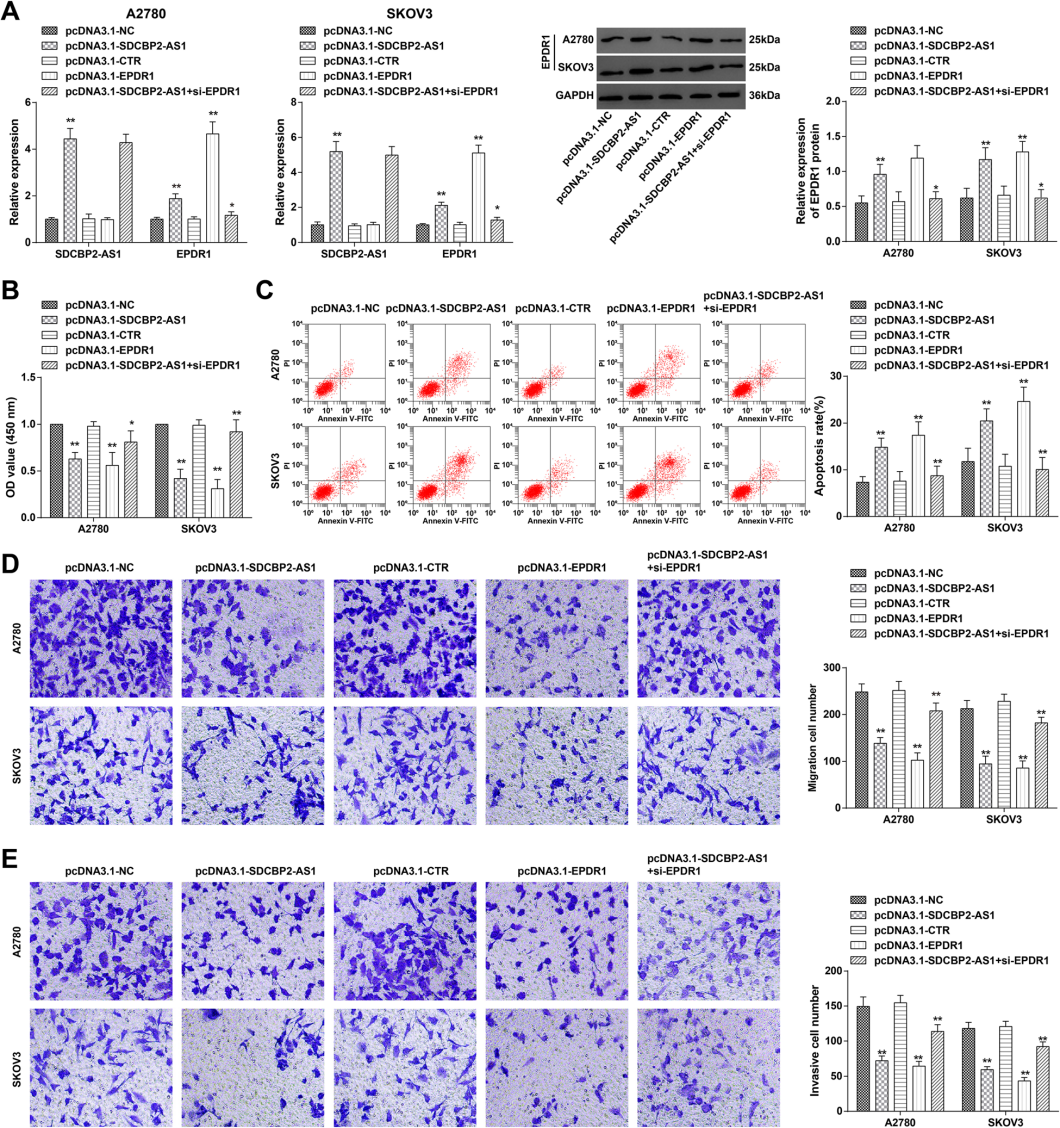
Upregulating lncRNA SDCBP2-AS1 or EPDR1 suppresses OC cell development. **A** RT-qPCR and Western blot analysis of lncRNA SDCBP2-AS1 and EPDR1 after transfection; **B** cell viability reflected by MTT assay; **C** cell apoptosis reflected by flow cytometry; **D** cell migration reflected by Transwell assay; **E** cell invasion reflected by Transwell assay; * *P* < 0.05, ** *P* < 0.01. Repetition = 3. Data were expressed in the form of mean ± standard deviation and analyzed by t test, one-way analysis of variance, and Tukey’s multiple comparison test

To specify the role of lncRNA SDCBP2-AS1 and EPDR1 in OC, pcDNA3.1-lncRNA SDCBP2-AS1 and pcDNA3.1-EPDR1 were utilized to upregulate lncRNA SDCBP2-AS1 and EPDR1 in OC cells (Fig. 2A). After that, cell viability, apoptosis, invasion, and migration were monitored by MTT, flow cytometry, and Transwell (Fig. 2B–E). Then, the findings manifested that in cells successfully upregulating lncRNA SDCBP2-AS1 or EPDR1, their viability, invasion, and migration were impaired while apoptosis rate was raised. However, si-EPDR1 transfection reversed the biological changes of OC cells caused by pcDNA3.1-lncRNA SDCBP2-AS1. Thus, it was indicated that upregulating lncRNA SDCBP2-AS1 or EPDR1 suppressed OC cell development.

### LncRNA SDCBP2-AS1 regulates EPDR1 through suppression of miR-100-5p

Considering the facts that lncRNA SDCBP2-AS1 and EPDR1 were downregulated and positively correlated in OC, overexpression of lncRNA SDCBP2-AS1 or EPDR1 inhibited OC development, and downregulation of EPDR1 reversed the effect of lncRNA SDCBP2-AS1 upregulation on OC; it was reasonably believed that lncRNA SDCBP2-AS1 functioned in OC by regulating EPDR1.

**Fig. 3:**
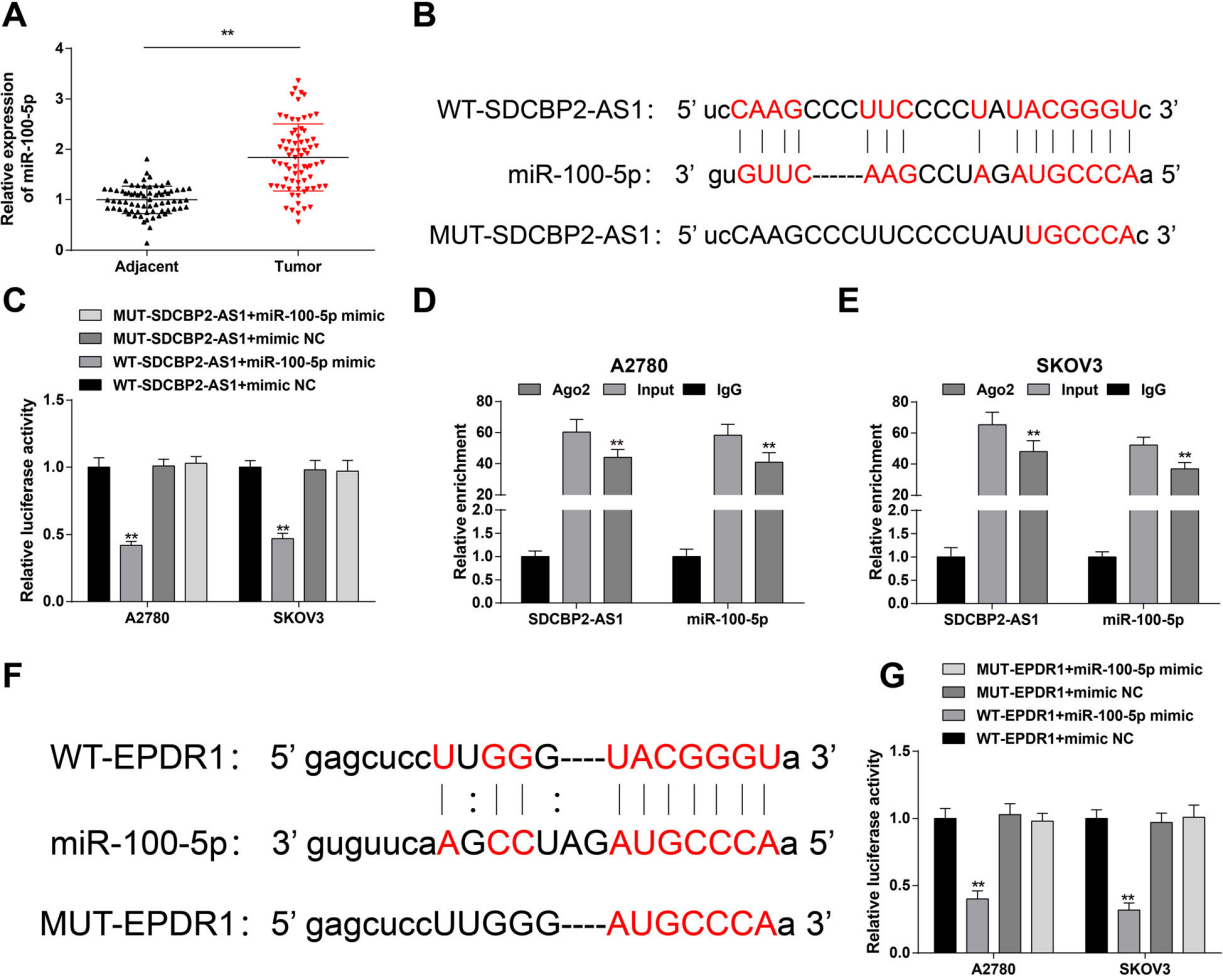
LncRNA SDCBP2-AS1 regulates EPDR1 through suppression of miR-100-5p. **A** RT-qPCR detection of miR-100-5p in OC tissues and adjacent tissues (*n* = 71); **B** starBase prediction of the binding sites between lncRNA SDCBP2-AS1 and miR-100-5p; **C** verification of the relation between lncRNA SDCBP2-AS1 and miR-100-5p reflected by dual-luciferase reporter assay; **D**/**E** verification of the relation between lncRNA SDCBP2-AS1 and miR-100-5p in A2780 and SKOV3 cells reflected by RIP assay; **F** bioinformatics database prediction of the binding sites between EPDR1 and miR-100-5p; **G** verification of the relation between EPDR1 and miR-100-5p reflected by dual-luciferase reporter assay; * *P* < 0.05, ** *P* < 0.01. Repetition = 3 (**C**–**G**); Data were expressed in the form of mean ± standard deviation and analyzed by t test, one-way analysis of variance, and Tukey’s multiple comparison test

In this study, we speculated that the regulation of EPDR1 by lncRNA SDCBP2-AS1 may be related to miRNA. Exactly, RT-qPCR detection tested miR-100-5p being upregulated in OC tissues (Fig. 3A). starBase predicted a specific binding region between lncRNA SDCBP2-AS1 and miR-100-5p (Fig. 3B). Luciferase activity detection revealed an impaired luciferase activity in cells co-transfected with miR-100-5p mimic and Wt-lncRNA SDCBP2-AS1 (Fig. 3C). In addition, RIP assay turned out that lncRNA SDCBP2-AS1 and miR-100-5p could be precipitated and raised by Ago2 antibody (Fig. 3D, E). In combination, lncRNA SDCBP2-AS1 was confirmed as a sponge for miR-100-5p.

The potential target genes of miR-100-5p were explored, and bioinformatics database software analysis predicted a binding region between miR-100-5p and EPDR1 (Fig. 3F). To confirm that, dual luciferase reporter gene test was performed which finally determined that miR-100-5p mimic inhibited the luciferase activity of Wt-EPDR1 in cells (Fig. 3G). Thus, EPDR1 was confirmed as a target of miR-100-5p.

Briefly, lncRNA SDCBP2-AS1 could regulate EPDR1 through sponging miR-100-5p.

### Elevating miR-100-5p mitigates the effect of upregulated lncRNA SDCBP2-AS1 on OC

**Fig. 4:**
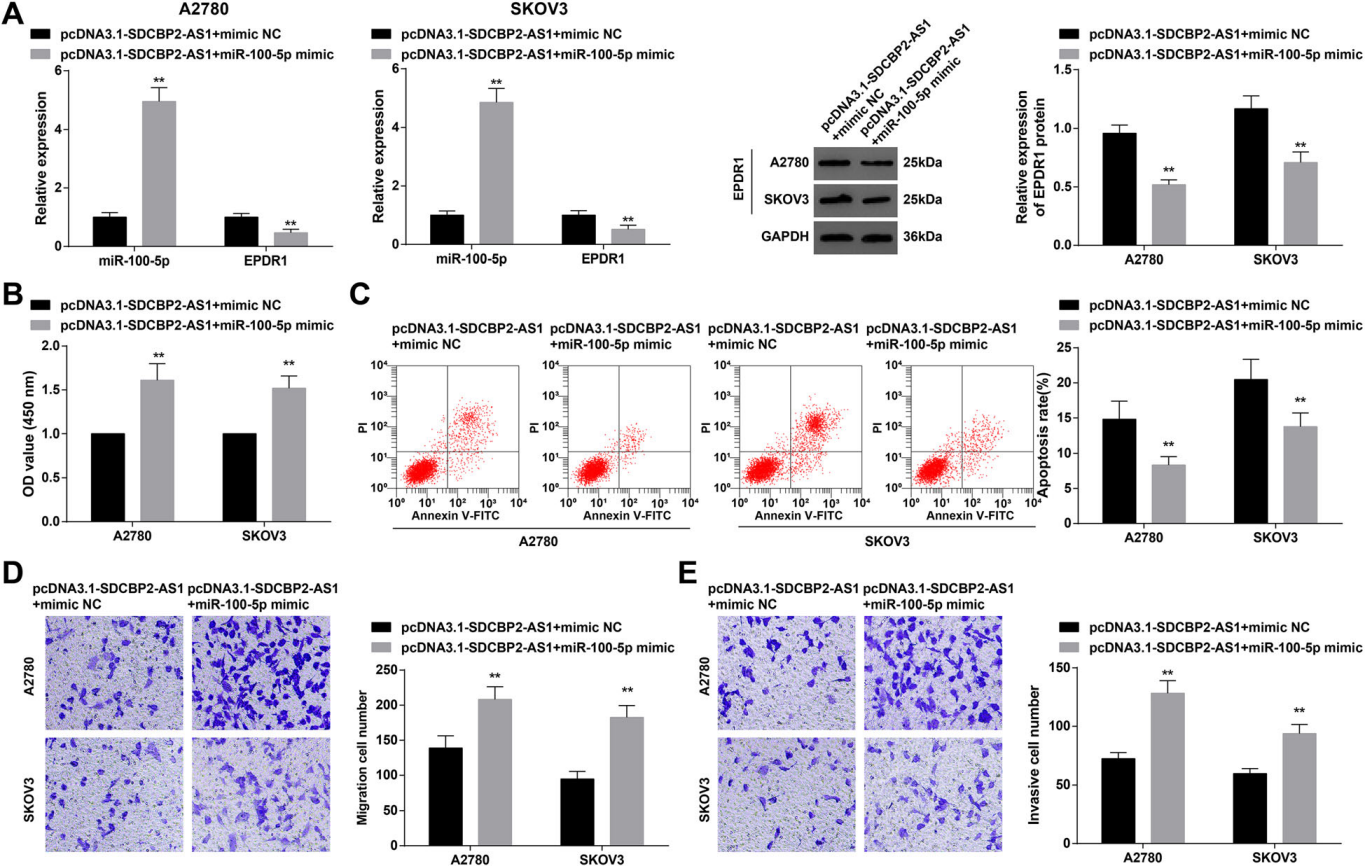
Elevating miR-100-5p mitigates the effect of upregulated lncRNA SDCBP2-AS1 on OC. **A** RT-qPCR and Western blot analysis of lncRNA SDCBP2-AS1, miR-100-5p, and EPDR1 after transfection; **B** cell viability reflected by MTT assay; **C** cell apoptosis reflected by flow cytometry; **D** cell migration reflected by Transwell assay; **E** cell invasion reflected by Transwell assay; * *P* < 0.05, ** *P* < 0.01. Repetition = 3. Data were expressed in the form of mean ± standard deviation and analyzed by t test

To identify the role of miR-100-5p in lncRNA SDCBP2-AS1 inhibiting OC, a rescue experiment was carried out. OC cells were transfected with pcDNA3.1-lncRNA SDCBP2-AS1 and miR-100-5p mimic. Then, through RT-qPCR and Western blot, pcDNA3.1-SDCBP2-AS was found to inhibit miR-100-5p and promote EPDR1 levels, while miR-100-5p mimic reversed pcDNA3.1-SDCBP2-AS-induced promotion to EPDR1 expression (Fig. 4A). Also, from cell biological functions to see, miR-100-5p mimic reversed pcDNA3.1-SDCBP2-AS-induced suppression to OC cell progression (Fig. 4B–E). Therefore, it was believed that lncRNA SDCBP2-AS1 regulated EPDR1 through miR-100-5p to inhibit OC development.

## Discussion

OC is a heterogeneous disease, and EOC has the highest mortality rate in gynecological cancers [23]. Our experimental research provided a glimpse of the molecular mechanism of OC from lncRNA SDCBP2-AS1 perspective. At first, we successfully measured the downregulated lncRNA SDCBP2-AS1 and EPDR1 in OC tissues and cells which were implicated to involve in OC occurrence and development. Also, we observed that upregulation of lncRNA SDCBP2-AS1 or EPDR1 in OC cells functioned to impede cell viability, invasion, and migration while raised apoptosis rate. Deeply, inhibition of EPDR1 would impair lncRNA SDCBP2-AS1 upregulation-mediated disturbance of OC progression. Subsequently, miR-100-5p was considered as the one that mediated the regulation of lncRNA SDCBP2-AS1 on EPDR1. Finally, re-expression of miR-100-5p in OC cells overexpressing lncRNA SDCBP2-AS1 was proved to induce cell malignant phenotype. Thus, it could be summarized that lncRNA SDCBP2-AS1 delayed OC development through upregulating miR-100-5p-mediated EPDR1.

LncRNA SDCBP2-AS1 that regulates tRNA modification is one of the downregulated lncRNAs in thyroid cancer, and higher level of lncRNA SDCBP2-AS1 indicates favorable disease-free survival, partly suggesting its suppression on tumor growth [10]. In addition to human cancer, aberrant expression of lncRNA SDCBP2-AS1 has been determined in osteoporosis, manifesting a downregulation in osteoblastic cells [24]. Except for the two reported papers, less research has revealed the mechanism of lncRNA SDCBP2-AS1 in diseases.

Our study displayed a binding relation between lncRNA SDCBP2-AS1 and miR-100-5p, but little few study could further validate this finding. Deregulation of miR-100-5p has been recognized in illnesses, and targeting miR-100-5p may develop cellular tools to manage diseases. In our data analysis, miR-100-5p expression was enhanced in OC, which was accorded to a recent paper that implies miR-100 upregulation in serum exosomes of EOC patients [25]. miR-100-5p expression is promoted in OSCC and acute myeloid leukemia that is predictive of dismal prognosis [18, 26] and higher miR-100-5p serves as a potential biomarker in hepatocellular carcinoma and nephroblastoma [27, 28]. Hsa-miR-100-5p is examined to overexpress in human ovarian endometriotic stromal cells and hsa-miR-100-5p enhances invasion and motility of normal endometrial stromal cells [17]. In human cancers, such as RCC, miR-100-5p level is raised, and miR-100-5p introduced into RCC cells causes viability and migration promotion and apoptosis inhibition [20]. Similarly, colorectal cancer cells also manifest overexpressed miR-100-5p level that is regulated by lncRNA PGM5 antisense RNA 1, and miR-100-5p upregulation strengthens proliferation, migration, and invasion through lowering SMAD4 level in cells [29]. In nasopharyngeal carcinoma, an increment is suggested in miR-100-5p expression which is mediated by forkhead box protein A1, and depletion of miR-100-5p hampers the malignant behaviors of cancer cells whilst restoration of miR-100-5p exerts oppositely [12]. In addition to that, silencing of miR-100-5p in dormant prostate cancer cells creates a niche for aggrandizing apoptosis, hindering proliferation, and preventing drug resistance against castration [19]. Showing a consistency with previous papers, the pro-tumor effect of miR-100-5p was witnessed in OC as well.

miR-100-5p was evidenced to target EPDR1 in this research. Moreover, a positive correlation was observed between lncRNA SDCBP2-AS1 and EPDR1 in OC that asks further researches to confirm. EPDR1 mRNA expression presents an impairment in breast cancer, and breast cancer cells containing overexpressed EPDR1 are characterized by suppressed cell activities [22]. As mentioned in the present work and published paper, EPDR1 overexpression functions to degrade tumor progression.

## Conclusion

To sum up, the feedback loop of lncRNA SDCBP2-AS1, miR-100-5p, and EPDR1 has been partly comprehended in our paper, as mirrored by the theory that lncRNA SDCBP2-AS1 upregulates miR-100-5p-targeted EPDR1, and obstructs the aggressiveness of OC cells. This study has more or less widened our horizon to the potential treatments of OC.

## Data Availability

Not applicable

## Abbreviations

OC: Ovarian cancer
EPDR1: Ependymin-related protein 1
miR: MicroRNA
EOC: Epithelial OC
OSCC: Oral squamous cell carcinoma
RCC: Renal cell carcinoma
FBS: Fetal bovine serum
NC: Negative control
RT-qPCR: Reverse transcription quantitative polymerase chain reaction
RIP: RNA immunoprecipitation
ANOVA: Analysis of variance
